# The role of renal biopsy in a patient with multiple synchronous cancers: a case report

**DOI:** 10.1186/1757-1626-2-9350

**Published:** 2009-12-18

**Authors:** Andrey Kaprin, Pavel Nesterov, Andrey Fadeev

**Affiliations:** 1 Russian Scientific Center of Roentgen-Radiology of Rosmedtechnology, 86 Profsoyuznaya St, Moscow, 117997, Russia

## Abstract

A 51-year-old male with a long history of tobacco smoking presented to the outpatient clinic with left renal colic. A renal ultrasound revealed a mass in the left kidney. The patient was admitted to surgical clinic of Russian Scientific Center of Roentgen-Radiology of Rosmedtechnology. A renal biopsy and subsequent histopathological tests revealed adenocarcinoma of the right kidney of most likely metastatic origin. This discovery has lead to vigorous diagnostics search for the primary tumor. Finally, the following diagnosis was established: Primarily-multiple synchronous cancer: cancer of the left kidney T1N0M0, cancer of the thyroid gland T2N0M1, metastasis to the right kidney and lungs. The patient had left kidney and thyroid gland removed and was successfully treated with radioiodine therapy. The patient remains alive and well 7 months since his admission to our clinic. We report this case to emphasize the importance of the renal biopsy and thorough histological analysis, which made it possible to diagnose thyroid cancer in this patient.

## Introduction

Renal cell carcinoma is a widespread disease. It ranked at the 10^th ^place among malignant neoplasms in humans and is the third most prevalent malignant neoplasms of genitourinary system. The incidence of renal cancer is growing all over the world, especially in economically developed countries [1]. In Russia, renal cell carcinoma is the 5^th ^leading cause of death among all malignancies [2]. The most specific method for diagnosing any malignancy is biopsy with subsequent morphological study of the specimen. However, many clinics in our country do not perform renal biopsy at all and totally relay on imaging methods for diagnosing kidney cancers.

## Case presentation

A 51-year-old white male was presented to an outpatient clinic with left renal colic. A renal ultrasound revealed a renal mass in the left kidney. The patient was referred and admitted to surgical clinic of Russian Scientific Center of Roentgen-Radiology and Rosmedtechnology on June 03, 2008 with admission diagnosis: "Primary-multiple synchronous cancer, cancer of right kidney T2N0M0, cancer of left kidney T2N0M0".

The patient worked as an electronics engineer. He was not under any medical care and did not take any medications. He has a history of 20 pack-years of tobacco smoking. His regular alcohol consumption was about 10 standard drinks per week.

At initial presentation, the patient (height = 178 cm; weight = 94 kg; BP 130/80 mmHg, pulse 74 per minute, T-36.6°C) was awake and oriented and did not complain on any pain or discomfort. Peripheral lymph nodes were not unremarkable; skin was normal. Abdomen was soft, non-tender. Kidneys, liver, and spleen were not palpable. External genital organs: no changes. Per rectum: no changes. CBC, biochemical panel and UA were unremarkable. No changes on ECG and chest radiograph were noted.

**Figure 1:**
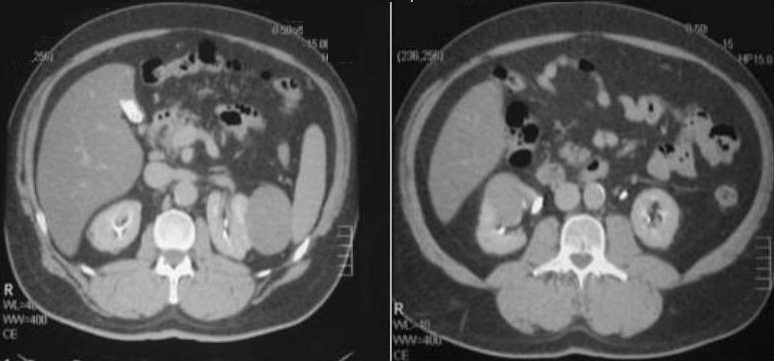
**Abdomen: CT scan**. Synchronous tumors of the kidneys.

**Figure 2:**
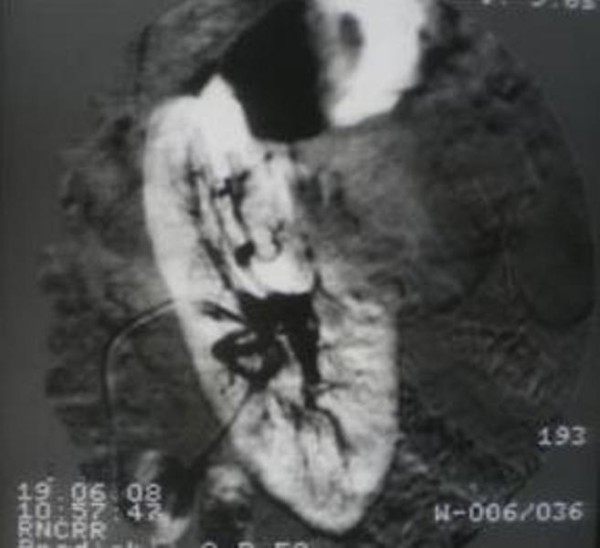
**Angiography: left kidney**. Tumor mass in the middle segment with mostly extrarenal location.

Contrast CT of the kidneys showed a hypoechogenic 80 × 60 mm mass with irregular margins in the middle-lower segment of the right kidney. The mass distorted major calyces, renal pelvis, and the contours of the right kidney and accumulated a contrast. A similar 70 × 55 mm mass was revealed in the upper segment of the left kidney with deformation of anterior-lateral contours of the left kidney and was adjacent to the spleen and to the upper calyx of the left kidney without involving the calyx. Calyces and renal pelvis of the left kidney were not enlarged (Figure 1 and Figure 2). No changes were noted in organs of abdominal cavity.

Ultrasound of the abdomen and kidneys yielded similar changes. Bilateral lesions of the kidneys and mostly extrarenal location of the lesion in the left kidney were crucial in determining a possibility of organ-saving operation on the left kidney.

Angiography revealed that each kidney is supplied with one main vessel at the level of upper margin of L_2_. After contrasting of the right renal artery in the middle and lower segments an oval mass was found with arcuate pressing of vessels in the lower segment of the right kidney. Selective contrasting of the left kidney revealed avascular mass on the external contour of the left kidney with deformation of the kidney contour in the upper and middle segments. Calyces and renal pelvises of both kidneys were unchanged.

Fine needle aspiration cytology of the renal masses showed renal cell carcinoma, clear cell type in the left kidney and renal cell carcinoma, possibly chromophobe type in the right kidney.

**Figure 3:**
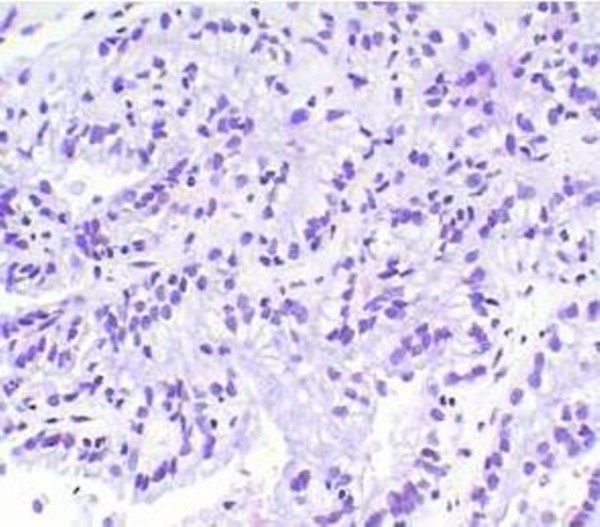
**A fragment of histological preparation of the left kidney biopsy**. Clear cell carcinoma with moderate nuclear polymorphism. Hematoxilin-Eosin stain ×200.

**Figure 4:**
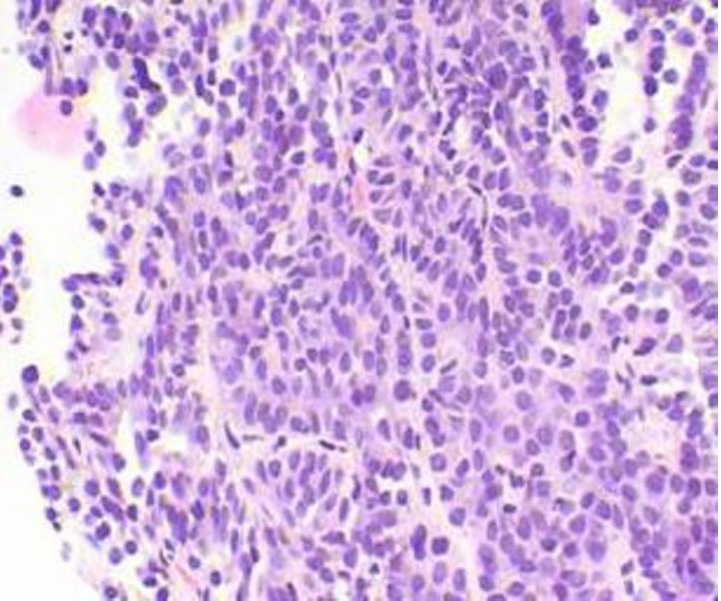


However, histological study of biopsy specimens concluded that the tumor of the right kidney is presented by adenocarcinoma that probably has metastatic origin; the tumor of the left kidney is renal cell carcinoma (Figure 3, 4).

Colonoscopy, esophagogastroduodenoscopy, ultrasonography of the thyroid gland and transrectal ultrasonography have been performed in order to locate the primary tumor. Ultrasonography of the thyroid gland showed that while the shape and size of the thyroid gland were normal, the structure of the gland was heterogeneous. Right lobe: a 12 mm node with distinct margins in the lower pole. Left lobe: two nodes 6 mm and 20 mm with distinct contours and two hypoechogenic nodes 7 mm and 14 mm with indistinct, rough contours and foci of calcification. Cervical lymph nodes were unremarkable. The first fine needle aspiration cytology of the described thyroid nodes showed erythrocytes, groups and layers of follicular epithelium with proliferation and dysplasia, colloid, connective tissue fragments, but no malignant cells.

In order to exclude a primary tumor of the lungs a CT scan of the chest has been performed. The CT scan showed metastatic lesions in both lungs. Serial CT with IV contrast revealed two lesions 5 mm in the S1 segment of the left lung; a 5 mm lesion in the S6 segment and a 2-3 mm lesion in S8 segment of the right lung. Lumens of the trachea and large bronchi were traced. Structures of the mediastinum were intact.

Considering specific ultrasound changes of the thyroid gland and absence of a primary tumor in other glandular organs, a second puncture biopsy of the thyroid gland has been performed. Cytology smears showed complexes of moderately polymorphic cells that constitute papillary structures of papillary cancer. Histological study showed that among abundance of erythrocytes there is a group of hemosiderophages and a complex of cells with polymorphic nuclei and single mitosis that represent papillary structures of papillary cancer.

**Figure 5:**
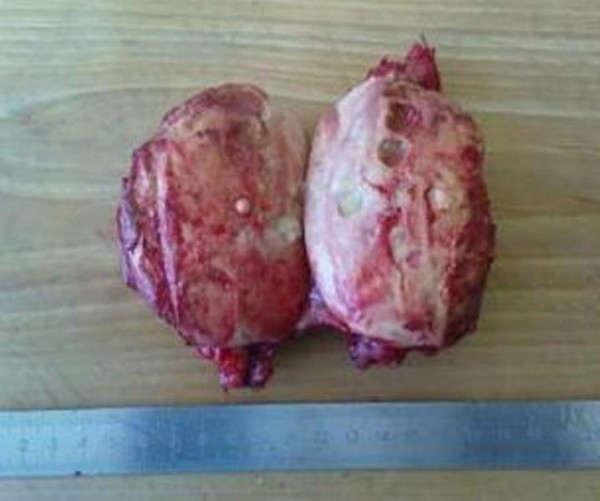
**Tumor of the left kidney 70 × 80 mm, removed within normal tissues**.

Thus, the final diagnosis has been established: "Primarily-multiple synchronous cancer: cancer of the left kidney T1N0M0, cancer of the thyroid gland T2N0M1, metastatic lesions of the right kidney and lungs". On the clinical conference following plan of the treatment has been approved: 1) a resection of the left kidney; 2) thyroidectomy; 3) performing an immunotherapy with subsequent radioiodine therapy. According to the plan of the treatment, the resection of the left kidney was performed on October 01, 2008. Histological study showed a malignant node that had renal cell carcinoma structure with clear cell and papillary components; the node was removed within normal tissues (Figure. 5).

The postoperative period proceeded without complications, the wound healed through primary intention; stitches were removed on 11^th ^day of hospitalization. The patient was discharged from the hospital on 12^th ^day.

Thyroidectomy was performed on December 11, 2008. On operation, enlargement of cervical lymph nodes up to 15 mm was found. The postoperative period proceeded without complications.

Thus, renal biopsy raised clinical suspicion for thyroid cancer and then helped to diagnose it. After iodine radiotherapy, the lesion in the right kidney has been stabilized in its growth. Lungs and bone metastases regressed. The size of cervical lymph nodes reduced. At present the patient feels well.

## Conclusion

The necessity of performing a renal biopsy in our country is considered equivocal and many clinics do not perform this procedure at all fully relaying on imaging diagnostic methods. However, the procedure is minimally invasive, ultrasound guided and often requires only local anesthesia. It also requires presence of a cytologist for urgent cytological study.

During the last 15 years we performed 762 renal biopsies. Following complications were encountered: hematoma in the site of the biopsy in 5.5% (42) of cases; gross hematuria in 1.5% (11) of cases; one case required an urgent nephrectomy because of massive bleeding from the tumor vessels. Injuries to adjacent organs and implantation metastases have not been noted.

## Abbreviations

BP: blood pressure; CBC: complete blood count; CT: computer tomography; ECG: electrocardiography; IV: intravenous; UA: urine analysis.

## Consent

Written informed consent was obtained from the patient for publication of this case report and accompanying images. A copy of the written consent is available for review by the Editor-in-Chief of this journal.

## Competing interests

The authors declare that they have no competing interests.

## Authors' contributions

KA operated the patient. NP performed one of the stages of surgical treatment of the patient. FA was an attending physician of the patient. All authors have read and have approved the final manuscript.
